# Emulsion and Microemulsion Systems to Improve Functional Edible Oils Enriched with Walnut and Pistachio Phenolic Extracts

**DOI:** 10.3390/foods11091210

**Published:** 2022-04-21

**Authors:** Giuseppe Fregapane, Cristina Cabezas Fernández, María Desamparados Salvador

**Affiliations:** Facultad de Ciencias y Tecnologías Químicas, Universidad de Castilla-La Mancha, Camilo José Cela 10, 13071 Ciudad Real, Spain; cristina.cabezas@alu.uclm.es (C.C.F.); amparo.salvador@uclm.es (M.D.S.)

**Keywords:** pistachio, walnut, phenolic extracts, functional oils, antioxidant capacity

## Abstract

The purpose of this research was to improve the properties of functional edible oils with potential health promoting effects, enriched with phenolic-rich extracts obtained from pistachio and walnut (5.1 and 27.4% phenolic contents respectively), by means of emulsion and micro emulsion systems. Stable water-in-oil (W/O) emulsions were obtained employing polyglycerol polyrhizinoleate (PGPR) as emulsifier (0.5, 2% H_2_O in oil), despite having a whitish and opaque appearance; transparent and stable microemulsions were prepared using proper proportion (e.g., 97:3) between the oily phase and the mixture of aqueous phase and emulsifiers (3:2 lecithin-distilled monoglycerides (DMG). Total polar phenolics contents ranging between 257 and 835 mg/kg were obtained in the novel functional edible oils’ formulations, reaching higher content using walnut as compared to pistachio extracts. Antioxidant capacity determined by the 2,2-diphenyl-1-(2,4,6-trinitrophenyl)hydrazyl (DPPH) method increased approx. 7.5 and 1.5 times using walnut and pistachio extracts respectively. An emulsion using gallic acid and a microemulsion employing hydroxytyrosol, two well-known antioxidants, were also studied to compare antioxidant capacity of the proposed enriched oils. Furthermore, the oxidative stability of these products—very relevant to establish their commercial value—was measured under accelerated testing conditions employing the Rancimat equipment (100 °C) and performing an oven test (at 40 °C for walnut oils and 60 °C for pistachio and refined olive oils). Rancimat oxidative stability greatly increased and better results were obtained with walnut (2–3 times higher) as compared to pistachio extract enriched oils (1.5–2 times higher). On the contrary, under the oven test conditions, both the initial oxidation rate constant and the time required to reach a value of peroxide value equal to 15 (upper commercial category limit), indicated that under these assay conditions the protection against oxidation is higher using pistachio extract (2–4 times higher) than walnut’s (1.5–2 times higher). Stable emulsions and transparent microemulsions phenolic-rich nut oils (250–800 mg/kg) were therefore developed, possessing a higher oxidative stability (1.5–4 times) and DPPH antioxidant capacity (1.5–7.5 times).

## 1. Introduction

The consumption of phenolic compounds with biological activity is quite variable due to their dissimilar contents in individual foodstuffs and food groups consumed in our diet, being nuts a good source of them [1,2]. Thus, a fine strategy to guarantee a desirable polyphenols intake along the diet could be to produce enriched edible oils possessing well-known phenolics contents and bioactivity. 

Nut extracts have not been employed to enriched vegetable oils (despite their proved biological activity, as discussed below) except in the previous publication of our research group, which is one of the remarkable novelties of the reported research. Virgin pistachio, virgin walnut, and refined olive (practically phenolics free) oils were enriched using lyophilized phenolic-rich nuts extracts [3]. An inadequate improvement of the oil phenolic content was obtained by the straight addition of the nut extracts due to their polarity, and therefore the use of an emulsifier was necessary, as also reported in literature by Suarez et al. [4]. However, W/O emulsion systems using lecithin or Span 80 resulted also not satisfactory stables with time, giving rise to phase separation and moreover a fine dispersion and a homogeneous drop size was not reached. For this reason, a study of different emulsion and microemulsion systems has been carried out in this research with the goal to improve the properties of functional edible oils enriched with pistachio and walnut phenolic extracts.

Some nutrition interventional research, including the renowned PREDIMED (prevention with Mediterranean Diet), have revealed that an elevated phenolic intake from virgin olive oil or nuts is related to a reduced incidence of metabolic syndrome, cardiovascular diseases, cancer and age-related cognitive decline [5,6,7,8]. Moreover, rich-polyphenol virgin olive oils (VOO) have been shown to improve antioxidant and anti-inflammatory effects and to reduce the proliferation of cell adhesion molecules compared with low-polyphenol VOO [9,10,11].

Nowadays, nuts (pistachios, walnuts and almonds) are regarded as a basal component of a healthy diet; possessing an equilibrated mono- and polyunsaturated fatty acids profile, and containing other micronutrients, besides a diversity of bioactive compounds, with antioxidant and anti-inflammatory properties, that can beneficially impact health outcomes [12,13,14,15]. Indeed in July 2003, the United States Food and Drug Administration (FDA) approved the first qualified health claim specific to nuts and the risk of heart disease, quoting that “*scientific evidence suggests but does not prove that eating 1.5 ounces (42.5 g) per day of most nuts, as part of a diet low in saturated fat and cholesterol may reduce the risk of heart disease*”. Evidence suggests that nuts can lower low-density lipoprotein-cholesterol levels and hence, reduce the risk of coronary heart disease [16]. The mentioned PREDIMED as well as other clinical and epidemiological trials, have corroborated it. Moreover, they have also pointed out that an elevated consumption of nuts can decrease the incidence of metabolic syndrome, diabetes, hypertension, inflammatory conditions cancer, and total mortality [17,18]. 

The purpose of this study is therefore to improve the properties of functional edible oils with potential health promoting effects, enriched with phenolic-rich extracts obtained from pistachio and walnut, described in our previous research [3], by means of emulsion and microemulsion systems. 

## 2. Materials and Methods

### 2.1. Pistachio and Walnut Extracts and Their Enriched Oils

Samples of walnut (‘Lara’ cv.) and pistachio (‘Sirora’ cv.) (3 kg each) were provided by the Regional Research Centre Centro de Mejora Agraria El Chaparrillo (Ciudad Real, Spain) and by the firm Nueces de Navarra (Navarra, Spain). 

Walnut and pistachio phenolic-rich extracts were obtained as previously reported [3]. Briefly, 5 g of milled whole kernel and 20 mL solvent (80:20 *v*/*v* ethanol:water) were shacked for 10 min. Extending the shaking time or using an ultrasound assisted extraction no differences were practically obtained. The water-ethanol binary mixture was selected for being GRAS (Generally Recognized as Safe) since the extracts are going to be employed in foodstuffs. A vacuum concentrator (miVac Duo, Genevac Ltd., Ipswich, UK) was used to evaporate the supernatants and finally freeze-dried (Cryodos-45, Telstar, Terrassa, Spain) giving to the production of phenolic-rich nut extract powders. 

Virgin walnut oil (VWO) and virgin pistachio oil (VPO) obtained in this research—beside a refined olive oil (ROO; supplied by Aceites Toledo SA, Los Yebenes, Toledo, Spain), with a very low phenolics content (50 mg/kg and 0.4 mmol/kg DPPH)—were enriched using the pistachio and walnut extracts described above (Pex and Wex; containing 5.1 and 27.4% phenolics, respectively) [3]. The pistachio and walnut cold-pressed oils were extracted using a screw press (Komet Screw Oild, Expeller CA59G-CA563, IBG Monforts Oekotec GmbH & Co. KG, Mönchengladbach, Germany) employing a nozzle of 6 mm of diameter and a screw speed of 30 rpm [19,20]. 

An inadequate improvement of the oil phenolics content was obtained by the straight addition of the nut extracts due to their polarity, and therefore the study of an appropriate emulsifier system was necessary.

### 2.2. Emulsions and Microemulsions Systems

Several emulsifiers and co-surfactants have been used in order to prepare W/O emulsions and microemulsions: soy lecithin (Emulpur, Cargill, Barcelona, Spain), Span 80 (Sigma, Darmstadt, Germany), distilled monoglycerides (DMG; Palsaagrad, Juelsminde, Denmark), propylene glycol (Sigma, Darmstadt, Germany) and Polyglycerol Polyrricinoleate (PGPR; Palsaagrad, Juelsminde, Denmark)—a powerful hydrophobic emulsifier, produced by the esterification of fatty acids from castor oil, widely used in the food industry [21]. 

Emulsions were prepared by adding from 0.5 up to 4.0% of the resolved freeze-dried walnut or pistachio extract (100–250 mg/mL in ethanol:water 1:1) to the oil with 0.1–0.5% of emulsifier, using an ultrasound probe for 30 s in a thermostatized bath at 4 °C.

The preparation of functional oil W/O microemulsions rich in phenolic compounds was carried out according to the procedure proposed by Chatzidaki et al. [22]. The apolar edible oil (VPO, VWO or ROO) was initially mixed with the surfactants (1–10%)—a 3:2 lecithin/DMG (distilled glycerin monostearate) mixture—and left for about 12 h in agitation. Then, the aqueous phase (0.5–4.0%; milliQ water containing 100–250 mg/mL of the freeze-dried extract and 30% of propylene glycol) was dropwise added under stirring. 

### 2.3. Total Polar Phenolic (TPP) Analysis

Total polar phenolic (TPP) concentration was measured using the method reported by Gutfinger [23]. Concisely, 0.4 g of milled pistachio whole kernel was extracted twice in 20 mL MeOH:H_2_O:HCOOH (80:20:0.1, 10 + 10 mL), vortexing 2 min, using ultrasound (5 min) and finally centrifugated (10 min at 2000× *g*). Extracts were then filtered prior to analysis. 100–500 µL aliquots of the polar extracts were delivered into a 10 mL volumetric flask adding up to 8 mL water and 0.5 mL of the Folin-Ciocalteu reagent. The solution absorbance was read after 3 min at 725 nm against a blank solution using a UV-visible spectrophotometer (Agilent Technologies 8453). Gallic acid (GAE; between 10 and 120 mg/L), as external standard, was employed for the calibration curve. 

### 2.4. Determination of Individual Phenolic Compounds

Individual phenolic compounds were measured using an HPLC-DADESI-MS/MS system as previously reported [3]. Briefly, the analysis was carried out on an Agilent 1100 series system (Agilent, Waldbronn, Germany) equipped with a photodiode array detector (DAD) and an LC/MSD Trap VL electrospray ionization mass spectrometry (ESI-MS/MS), both coupled with an Agilent ChemStation for data processing. Aliquots of phenolic extracts, obtained as described in Section 2.1, were evaporated in a rotary evaporator at 35 °C under vacuum and were eventually dissolved in 200 μL of methanol/water (20:80, *v*/*v*) by being sonicated (5 min) and vortexed (2 min) before injecting 20 μL in the system. Quantification of pistachio phenolics was performed using the DAD chromatograms recorded at 280 nm (flavanols, flavanones and phenolic acids), 360 nm (flavonols) and 520 nm (anthocyanins) using specific calibration curves [3].

### 2.5. Total Antioxidant Capacity (TAC) Measurements

Total antioxidant capacity (TAC) has been determined by the 2,2-diphenyl-1-(2,4,6-trinitrophenyl)hydrazyl (DPPH) radical assay. The radical scavenging effect of the methanolic extract towards the synthetic radical DPPH was performed as reported previously [24]. Concisely, the polar extract (100 µL; obtained as mentioned in the previous point) was incorporated to a methanolic DPPH solution (2.9 mL, 6 × 10^−5^ M) and kept for 30 min in the dark. The decline in absorbance was then read at 515 nm employing an Agilent 8453 spectrophotometer. Trolox ((+/−)-6-hydroxy-2,5,7,8-tetra-methylchromane-2-carboxylic acid; between 0.2 to 0.9 M), as external standard, was employed to plot the calibration curve.

### 2.6. Peroxide Value

Peroxide value (PV, meq O_2_/kg) was measured following the European Regulation EEC 2568/91 (1991) and following amendments.

### 2.7. Oxidative Stability

Stability was measured by induction period (hours) of the oxidation determined by the Rancimat equipment (model 743 Metrohm Co., Basel, Switzerland) employing 3.0 g of oil heated to 100 °C and using 10 L/h air flow of according to ISO 6886:2016.

### 2.8. Accelerated Shelf-Life Testing (ASLT)

The emulsions and microemulsions obtained from enriched virgin nut oils and refined olive oil were stored at 40 °C (VWO) and 60 °C (VPO and ROO) in a heating chamber (Binder, Tuttlingen, Germany; model WTB). An orbital shaker was placed inside the chamber to keep the samples in agitation and thus facilitate homogeneous oxidation throughout the oil.

### 2.9. Statistical Analysis

ASLT assays were carried out in triplicate and the XLSTAT 19.5 statistical package (Addinsoft, Paris, France) was employed for the analysis of variance (ANOVA) and for the Fisher’s LSD multiple comparison test to analyze significant differences between the studied oil formulations in emulsion and microemulsion on the basis of the experimental data obtained (i.e., phenolic content, DPPH, OSI, etc.) as reported in tables.

## 3. Results and Discussion

### 3.1. Properties of the Nut Kernels and Their Phenolic-Rich Extracts

The walnut (‘Lara’ cv.) and pistachio (‘Sirora’ cv.) nuts used in this research as well as their lyophilized extracts were previously thoroughly studied by our research group [19,20,25].

In Table 1 the main characteristics of the nuts’ kernels and their phenolic-rich extracts are shown. Pistachio and walnut are avowed for a great polar phenolic content, 7 g/kg and 11 g/kg respectively (150% higher in walnut) in the case of ‘Sirora’ and ‘Lara’ in this research. The related determined antioxidant capacity (DPPH) is greater (530%) in walnut (0.106 mol/kg Trolox) than in pistachio (0.020 mol/kg Trolox).

Since the polar phenolic compounds of pistachio and walnut are rather polar, only a small quantity of them is solubilized in the oily matrix (9–14 mg/kg; [19,20], but similar to other seed virgin oils, such as soybean, sunflower, rapeseed and corn (10–40 mg/kg) [26]. Indeed, one of the chief motivations to study the suitability of enriching oils with the phenolics components from the same nut is exactly their relatively low level in the virgin oils in comparison to their kernels. 

The full characterization of the phenolic-rich extracts from ‘Lara’ and ‘Sirora’ nut varieties, determined by an HPLC-DADESI-MS/MS method, has also been previously reported [3]. These extracts showed much higher content in TPP: 51 g/kg, 700% greater than the related pistachio kernel; and 274 g/kg, 2200% greater than its walnut kernel. The TAC determined by the DPPH method, showed great activity: 255 mol Trolox/kg and 13 mol Trolox/kg (1500 and 630 times greater than their kernels) for walnut and pistachio respectively (Table 1). 

Contents of phenolic compounds main families in the kernel of ‘Sirora’ pistachio and its extract are also depicted in Table 1: almost 90% of the phenolic components found in pistachio belong to the flavanols family, both in extract and kernel, where procyanidins (6.3 g/kg in the extract) are richer than catechins (4.1 g/kg) and epicatechins (2.4 g/kg). On the other hand, the major phenolics measured in walnut kernel and its extract belongs to the family of hydrolysable tannins (2.1 in kernel and 92 g/kg in its extract), reaching approximatively 70% of total, similarly to data published by Slatnar et al. [27]. Flavanols and flavonols are also important families, being procyanidins (0.8 and 66 g/kg in kernel and in its extract) and catechins (18 g/kg in their lyophilized extract) the most relevant components of flavanols, in agreement with Slatnar et al. [27]

These great antioxidant capacities make them candidates of interest to assay their potential as ingredient with biological activity. In fact, a previous work of our group has described results of interest on the pistachio extract in MCF-7 cells [28]; leading to a relevant decline in the viability of MCF-7 breast cancer. Potential role of walnut consumption against diseases also has been recently described in literature [29].

### 3.2. Stability of Emulsions and Microemulsions Systems

As anticipated, an unsatisfactory enrichment in nuts’ phenolic components into the oil matrix (due to their polarity) was observed. For this reason, a study of different emulsion and microemulsion systems has been carried out in this research with the goal to improve functional edible oils enriched with lyophilized pistachio and walnut phenolic-rich extracts.

Preliminary study of emulsions using lecithin, Span 80 or DMG (distilled glycerin monostearate) showed that these systems were not satisfactorily stable with time, giving rise to phase separation in few days or weeks and moreover a good dispersion and a homogeneous drop size was not reached. 

On the contrary, employing PGPR (polyglycerol polyrhizinoleate) as emulsifier resulted in a much better emulsion stability, as also previously observed [21]. Indeed, W/O emulsions with 0.5% PGPR and 2% distilled H_2_O in refined olive oil (ROO), despite having a whitish and opaque appearance, showed a remarkable stability over time and moreover a homogeneous size and adequate dispersion (by observation under microscope with 100 increases; data/images not reported). 

The use of a microemulsion has a double objective: on one hand, to obtain transparent emulsions that have a similar appearance to the base oil of which they are formed (one of the main goals of this study) and on the other, that the microemulsions are stable in time and at room temperature, that is, no phase break occurs.

**Figure 1:**
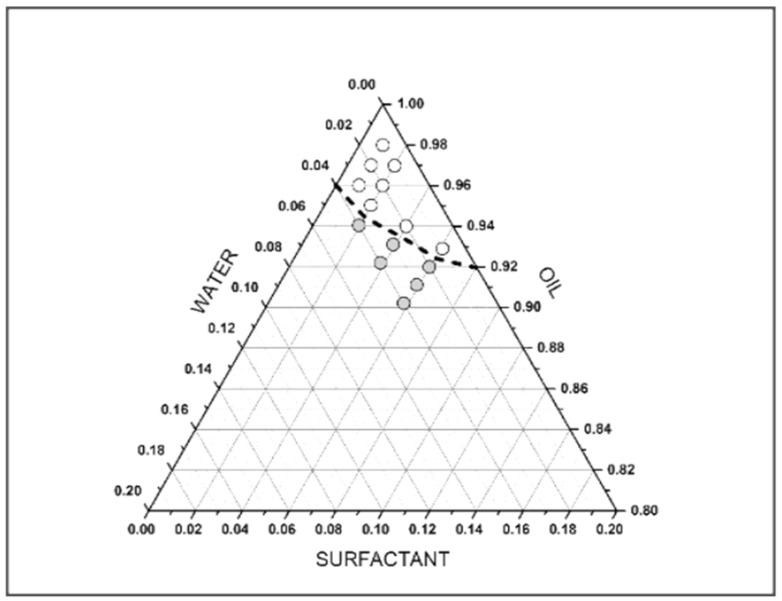
Properties of formulated microemulsions with different oil, water and surfactant ternary proportions. white dots represent transparent microemulsions, while grey once correspond to opaque-looking ones.

Different microemulsion systems were formulated by varying the proportions of the aqueous, oily and surfactant phases in order to determine the proportions that allow to obtain transparent and stable microemulsions, as depicted in the ternary diagram reported in Figure 1, where the white dots represent the transparent microemulsions, while the grey once correspond to the opaque-looking. After these preliminary assays, it was decided to use a 97:3 proportion between the oily phase and the mixture of aqueous phase and emulsifiers, so as to use as little water as possible (1%) and thus enriching the oil with antioxidants modifying minimally the initial composition of the product and obtaining a transparent emulsion.

### 3.3. Phenolic Content and Antioxidant Capacity of Novel Oil Formulations

As previously stated, no enrichment of edible oil with phenolic extracts from nut has been reported, except in our previous paper [3]. Among various extraction/preparation and enrichment protocols assayed the solid-liquid extraction (SLE) reaches greater yields. The general procedure for edible oils augment usually take place in two main phases: (i) the extraction of the desired components from the raw material, for example leaves, herbs or nuts, as in this experimental work, and then (ii) the enrichment of the oil with the obtained extract [30].

Virgin walnut oil (VWO) and virgin pistachio oil (VPO) obtained in this research, besides a refined olive oil (ROO), with very low phenolics (50 mg/kg TPP and 0.4 mmol/kg DPPH) and used as control, were enriched using lyophilized pistachio and walnut phenolic-rich extracts (abbreviated as Pex and Wex) according to the procedure reported previously in the material and method section of this article.

The properties of the novel phenolic-enriched oils obtained using PGPR emulsion and lecithin-DMG microemulsion are reported in Table 2. A wide range of TPP (total polar phenolics) concentrations ranging between 257 and 835 mg/kg have been obtained in the new oil formulations prepared. A higher TPP content has been obtained using walnut extracts (Wex: 685, 835 and 678 mg/kg in VWO, VPO and ROO respectively in emulsion; Table 2) as compared to pistachio extracts (Pex: 310, 257 and 316 mg/kg, respectively). This is mainly due to the different phenolic content in the two nut type extracts used. 

Under microemulsion conditions a lower phenolic-enrichment was apparently obtained: e.g., 426 vs. 685 mg/kg in VWO-Wex, 238 vs. 310 mg/kg in VWO-Pex or 499 vs. 678 mg/kg in ROO-Wex; Table 2).

An emulsion using gallic acid (approx. 100 mg/kg; VWO-gal) and a microemulsion employing hydroxytyrosol (approx. 500 mg/kg; VWO-htyr), two well-known antioxidants, has been as well prepared with the purpose of comparing antioxidant capacity and stability given to the oil formulations (Table 2).

A great improvement in the antioxidant capacity (DPPH), as compared to the base oil, was obtained in all of the enriched oils produced: e.g., 4.98 vs. 0.11 mmol/kg in emulsionated VWO-Wex and WVO; 2.89 vs. 0.09 mmol/kg in microemulsionated VWO-Wex and VWO; 0.36 vs. 0.07 in emulsionated VPO-Pex and VPO; or 5.30 vs. 0.13 mmol/kg in emulsionated ROO-Wex and ROO (Table 2). The enrichment of ROO resulted in an antioxidant capacity nearby the related enriched VWO or VPO (5.30 and 0.44 mmol/kg Trolox using Wex and Pex respectively). 

Taking into account that the phenolic content greatly differs in the oil formulations studied, the relationship between the values of DPPH and phenolic content has also been reported in Table 2 (DPPH/TPP). This parameter was much higher when extract from walnut (Wex) were employed: 7.3, 7.6 and 7.8 for VWO, VPO and ROO spiked with Wex, as compared to 1.8, 1.4 and 1.4 when Pex was employed (Table 2), confirming once again the higher antioxidant capacity of the lyophilized walnut extracts (Wex) under this test conditions. 

### 3.4. Oxidative Stability of Studied Oil Formulations in Emulsion and Microemulsion

**Figure 2:**
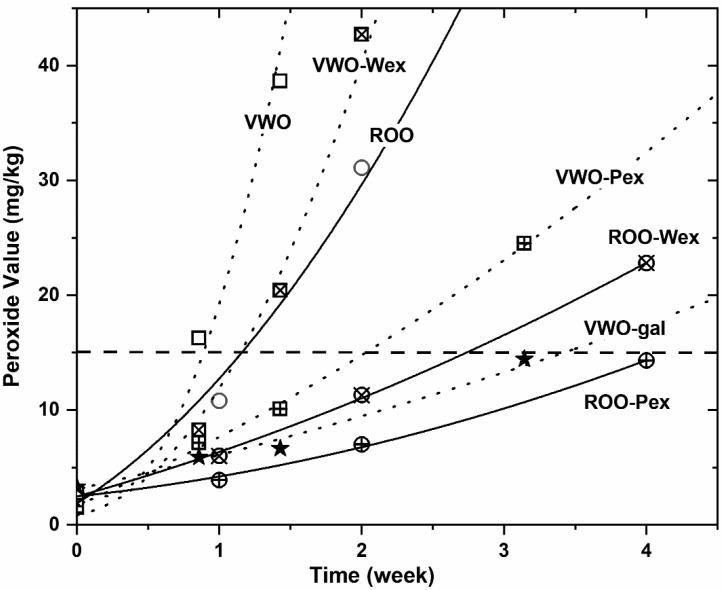
Evolution of the oxidation process of oil formulations under oven test * conditions. Oven Test: 40 °C in VWO; 60 °C in VPO and ROO. VWO, virgin walnut oil; VPO, virgin pistachio oil; ROO, refined olive oil. Wex, walnut phenolic extract; Pex, pistachio phenolic extract; gal, gallic acid.

The oxidative stability of the novel oil formulations is very relevant to establish the commercial value of the proposed enriched oils. It was measured under accelerated testing conditions employing the Rancimat equipment (100 °C, air flow 10 L/h) and performing an Oven Test (at 40 °C for VWO and 60 °C for VPO and ROO) as reported in Table 3 and Figure 2. 

VWO resulted the most sensitive to oxidation (5.3 h of induction period in emulsion and 6.7 h in microemulsions; Table 3), due to its great unsaturated fatty acid proportion (>12% linolenic and >60% linoleic). Its fortification with Wex and Pex greatly improved its low stability to oxidation (2.4-fold in emulsion and 1.5 in microemulsion, although in the latter system the TPP content was lower 426 vs. 685 mg/kg (Table 2). 

The stability of ROO and VPO measured by Rancimat was alike: 42.4 and 40.1 h respectively. The oils fortified with extracts from pistachios (Pex) augement their induction period a 13–60% (up to 68.8 and 52.4 h, respectively in emulsions). 

Finally, the stability to oxidation of fortified oils apparently resulted in higher values when extracts from walnut were used (Wex, e.g., 12.5, 68.8 and 121.8 h in VWO, VPO and ROO in emulsions; Table 3) as compared to pistachio’s (Pex: 10.5, 52.4, 68.8 h, respectively; Table 3), revealing once more that phenolic-rich extracts from walnut (Wex) evidently own greater antioxidant activity than pistachio (Pex). 

Nevertheless, measuring the oxidative stability by means of an oven test (at 40 °C for VWO and 60 °C for VPV and ROO), both the initial oxidation rate constant (K, meq O_2_/kg/day) and the time (day) required to reach a value of peroxide value (PV) equal to 15 (upper commercial category limit; PV15), indicate that the protection against oxidation is higher using pistachio extract (Pex) than walnut’s (Wex; e.g., 14.7 vs. 8.7, 6.3 vs. 4.9 and 29.1 vs. 19.3 days to reach PV = 15 for VWO, VPO and ROO, respectively; Table 3 and Figure 2). In microemulsion that difference is apparently lower (e.g., 19.7 vs. 17.9 for VWO).

The activity of VWO-gal (approx. 100 mg/kg gallic acid added) in emulsion showed a higher antioxidant effect than Wex and Pex enriched oils both under Rancimat and oven test conditions, since a lower concentration of this well-known antioxidant gave a higher induction period (16.5 h) as well as a lower initial oxidation rate (K; 0.30 meq/Kg/d) and therefore a longer time to reach PV = 15 (PV15; 39.5; as depicted in Table 3 and Figure 2). VWO-htyr (approx. 500 mg/kg) in microemulsion showed a similar behavior: higher Rancimat induction period (14.2 h), lower K (0.14 meq/Kg/d) and longer PV15 (71.9 d) than the other enriched oil formulaitons; although in this case the concentration of hydroxytyrosol in VWO-htyr was higher than VWO-Wex and VWO-Pex (538, 426 and 238 mg/kg respectively as reported in Table 2).

The protective effect against oxidation of Pex and Wex addition to the vegetable oil base is clearly visible in Figure 2, where the evolution of the oxidation process under oven test assay conditions (40 °C for VWO and 60 °C for ROO and VPO) is plotted. Indeed, the addition of phenolic-rich nut extracts (Wex or Pex) to VWO or ROO clearly increased their stability, reducing the rate of the peroxide formation and therefore increasing the time required to reach a PV = 15. In both cases, as already mentioned, the enrichment with pistachio extract (Pex) resulted in a better oxidation stability performance of the oil formulations under this ASLT (accelerated shelf-life testing) conditions, giving to the ROO-Pex enriched oil the best stability (Figure 2). It is very important to recall that the oxidation mechanism is different at mild oxidation conditions (oven test) than at higher temperature, as in the Rancimat apparatus, explaining the different performance observed using Wex and Pex under these two different testing conditions.

## 4. Conclusions

These novel functional edible oils possessing a well-defined phenolics amount and profile, as well as biological activity, may represent a fine strategy to guarantee an optimal polyphenols consumption, since the intake of phenolics with bioactivity considerably vary due to their distinctive contents in the various foodstuffs of the diet. 

In this experimental work, stable emulsions (PGPR) and even transparent microemulsions (lecithin-DMG) systems were developed, allowing the formulation of phenolic-rich products (from 250 to more than 800 mg/kg), possessing a higher oxidative stability (1.5–4 times) and DPPH antioxidant capacity (1.5–7.5 times) as opposed to the straightforward incorporation of extracts from nut, which produces an unsatisfactory enrichment with extracted phenolic components due to their polarity. Moreover, W/O emulsion systems using lecithin or Span 80 resulted not stables with time.

## Figures and Tables

**Table 1:** Total polar phenolic (TPP) content, antioxidant activity (DPPH) and main individual phenolic families’ contents * in walnut (‘Lara’ cv.) and pistachio (‘Sirora’ cv.) nut kernels and their corresponding lyophilized phenolic-rich extracts (*n* = 3).

	Walnut	(‘Lara’ cv)	Pistachio	(‘Sirora’ cv.)
	Kernel	Extract	Kernel	Extract
TPP (g/kg gallic acid)	11 ± 1 ^b^	274 ± 4 ^d^	7 ± 1 ^a^	51 ± 3 ^c^
DPPH (mol/kg Trolox)	0.11 ± 0.02 ^b^	255 ± 3 ^d^	0.020 ± 0.01 ^a^	13 ± 1 ^c^
Flavanols (g/kg)	0.9 ± 0.1 ^a^	84 ± 1 ^d^	2.3 ± 0.3 ^b^	12.7 ± 0.3 ^c^
- *procyanidins*	0.8 ± 0.1 ^a^	66 ± 1 ^c^	1.1 ± 0.1 ^a^	6.3 ± 0.3 ^b^
- *catechins*	0.2 ± 0.1 ^a^	18 ± 1 ^d^	0.7 ± 0.1 ^b^	4.1 ± 0.2 ^c^
- *epicatechins*	nd	nd	0.6 ± 0.1 ^a^	2.4 ± 0.2 ^b^
Flavonols (g/kg)	0.9 ± 0.1 ^c^	84 ± 1 ^d^	0.1 ± 0.0 ^a^	0.3 ± 0.0 ^b^
Flavanones (g/kg)	nd	nd	nd	0.1 ± 0.0
Anthocyanins (g/kg)	nd	nd	0.1 ± 0.0 ^a^	0.9 ± 0.0 ^b^
Hydrolysable Tannins (g/kg)	2.1 ± 0.4 ^a^	92 ± 1 ^b^	nd	nd
Ellagic acid deriv. (g/kg)	0.1 ± 0.0 ^a^	13 ± 1 ^b^	nd	nd

* determined by an HPLC-DADESI-MS/MS. ^a–d^, a different superscript letter means a statistically significant difference (at 95%) between data in rows within the same variable (i.e., TPP, DPPH, flavanols, …). nd, not determined.

**Table 2:** Phenolic content and DPPH antioxidant capacity of studied of oil formulations in emulsion and microemulsion (*n* = 3).

	Emulsion			Microemulsion		
	TPP (mg/kg)	DPPH (mmol/kg)	DPPH/TPP	TPP (mg/kg)	DPPH (mmol/kg)	DPPH/TPP
Walnut Oil						
VWO	12 ± 1 ^a^	0.11 ± 0.01 ^a^		10 ± 1 ^a^	0.09 ± 0.01 ^a^	
VWO-Wex	685 ± 28 ^d^	4.98 ± 0.62 ^d^	7.3	426 ± 36 ^d^	2.89 ± 0.24 ^c^	6.8
VWO-Pex	310 ± 15 ^c^	0.55 ± 0.07 ^b^	1.8	238 ± 32 ^c^		
VWO-gal	133 ± 2 ^b^	1.28 ± 0.04 ^c^	9.6	101 ± 12 ^b^	1.54 ± 0.23 ^b^	15.3
VWO-htyr				538 ± 41 ^e^		
Pistachio Oil						
VPO	18 ± 2 ^a^	0.07 ± 0.01 ^a^				
VPO-Wex	835 ± 42 ^c^	6.37 ± 0.73 ^c^	7.6			
VPO-Pex	257 ± 34 ^b^	0.36 ± 0.05 ^b^	1.4			
Refined Oil						
ROO	30 ± 2 ^a^	0.13 ± 0.02 ^a^		24 ± 3 ^a^	0.09 ± 0.01 ^a^	
ROO-Wex	678 ± 63 ^c^	5.30 ± 0.48 ^c^	7.8	499 ± 56 ^c^	2.73 ± 0.45 ^b^	5.5
ROO-Pex	316 ± 42 ^b^	0.44 ± 0.06 ^b^	1.4	254 ± 24 ^b^		

VWO, virgin walnut oil; VPO, virgin pistachio oil; ROO, refined olive oil. Wex, walnut phenolic extract; Pex, pistachio phenolic extract; gal, gallic acid; htyr, hydroxytyrosol; TPP, total phenolic content (mg/kg as gallic acid); DPPH/TPP (mmol Trolox/g phenolics/kg oil). ^a–e^, a different superscript letter means a statistically significant difference (at 95%) between data in columns within the same oil type (walnut, pistachio and refined).

**Table 3:** Results of Rancimat and oven tests * of oil formulations in emulsion and microemulsion (*n* = 3).

	Emulsion			Microemulsion		
	OSI (h)	K (meq/kg/d)	PV15 (d)	OSI (h)	K (meq/kg/d)	PV15 (d)
Walnut Oil						
VWO	5.3 ± 0.3 ^a^	2.34 ± 0.38 ^c^	4.7 ± 0.6 ^a^	6.7 ± 0.8 ^a^	0.71 ± 0.05 ^e^	11.3 ± 1.6 ^a^
VWO-Wex	12.5 ± 1.8 ^b^	1.01 ± 0.23 ^b^	8.7 ± 0.7 ^b^	10.0 ± 1.3 ^c^	0.53 ± 0.06 ^c^	17.9 ± 2.1 ^b^
VWO-Pex	10.5 ± 1.1 ^b^	0.91 ± 0.10 ^b^	14.7 ±1.6 ^c^	8.4 ± 0.5 ^b^	0.28 ± 0.03 ^b^	19.7 ± 2.0 ^b^
VWO-gal	16.5 ± 0.7 ^c^	0.30 ± 0.05 ^a^	39.5 ±5.7 ^e^	10.9 ± 0.9 ^c^	0.45 ± 0.05 ^c^	23.8 ± 2.2 ^c^
VWO-htyr			24.2 ±3.6 ^d^	14.2 ± 1.6 ^d^	0.14 ± 0.02 ^a^	71.9 ± 8.9 ^d^
Pistachio Oil						
VPO	40.1 ± 5.3 ^a^	2.95 ± 3.22 ^c^	2.8 ± 0.3 ^a^			
VPO-Wex	68.8 ± 8.2 ^c^	1.70 ± 0.31 ^b^	4.9 ± 0.7 ^b^			
VPO-Pex	52.4 ± 4.8 ^b^	1.49 ± 0.23 ^a^	6.3 ± 0.5 ^c^			
Refined Oil						
ROO	42.4 ± 5.3 ^a^	2.03 ± 0.32 ^c^	7.4 ± 0.6 ^a^	44.9 ± 6.7 ^a^		
ROO-Wex	122 ± 17 ^c^	0.62 ± 0.05 ^b^	19.3 ± 2.4 ^b^	62.2 ± 8.3 ^c^		
ROO-Pex	68.8 ± 8.0 ^b^	0.34 ± 0.03 ^a^	29.1 ± 3.4 ^c^	50.6 ± 7.2 ^b^		

* 40 °C for VWO; 60 °C for VPO and ROO. VWO, virgin walnut oil; VPO, virgin pistachio oil; ROO, refined olive oil. Wex, walnut phenolic extract; Pex, pistachio phenolic extract. OSI, Rancimat (100 °C, 10 L/h) induction period (h); K, initial oxidation rate constant (meq O_2_/kg/day) and PV15, time (day) required to reach a value of peroxide value (PV) equal to 15 (upper legal/commercial category limit), at 40 °C (VWO) and 60 °C (VPO and ROO). ^a–e^, a different superscript letter means a statistically significant difference (at 95%) between data in columns within the same oil type (walnut, pistachio and refined).

## Data Availability

Data is contained within the article.

## Abbreviations

DPPH: antioxidant radical assay; gal, gallic acid; Pex, pistachio phenolic-rich extract; ROO, refined olive oil; TAC, total antioxidant activity; TPP, total phenolic content; VWO, virgin walnut oil; VOO, virgin olive oil; VPO, virgin pistachio oil; Wex, walnut phenolic-rich extract; W/O, water in oil.
